# An unexpected case of frontal headache: Silent corticotroph pituitary neuroendocrine tumor presenting as a sphenoid sinus mass

**DOI:** 10.1177/2050313X251332081

**Published:** 2025-03-29

**Authors:** Marie-Michelle McNicoll, Lamiae Himdi, Marc Tewfik, Salvatore Di Maio, Marie-Christine Guiot, Vincent Larouche

**Affiliations:** 1 Undergraduate Medical Education, McGill University, Montreal, QC, Canada; 2 Campus Outaouais, McGill University, Gatineau, QC, Canada; 3 Division of Otolaryngology and Head and Neck Surgery, Notre-Dame Hospital, Montreal, QC, Canada; 4 Division of Otolaryngology and Head and Neck Surgery, Department of Surgery, Université de Montréal, Montreal, QC, Canada; 5 Division of Otolaryngology and Head and Neck Surgery, McGill University Health Centre, Montreal, QC, Canada; 6 Division of Otolaryngology and Head and Neck Surgery, Department of Surgery, McGill University, Montreal, QC, Canada; 7 Division of Neurosurgery, Jewish General Hospital, Montreal, QC, Canada; 8 Department of Neurology and Neurosurgery, McGill University, Montreal, QC, Canada; 9 Division of Pathology, McGill University Health Centre—Montreal Neurological Hospital, Montreal, QC, Canada; 10 Departments of Pathology, Neurology and Neurosurgery, McGill University, Montreal, QC, Canada; 11 Division of Endocrinology and Metabolism, Jewish General Hospital, Montreal, QC, Canada; 12 Division of Endocrinology and Metabolism, Department of Medicine, McGill University, Montreal, QC, Canada

**Keywords:** Sphenoid sinus mass, pituitary neuroendocrine tumor, pituitary adenoma, transsphenoidal surgery, Cushing syndrome, corticotroph adenoma, silent corticotroph adenoma

## Abstract

We report the case of a 43-year-old male who presented with a 2-year history of frequent frontal headaches, initially attributed to sinus disease. Magnetic resonance imaging revealed a 30 mm mass in the right sphenoid sinus with extension into the cavernous sinus, encasing the internal carotid artery, and invading the sella turcica. Differential diagnosis based on imaging included sphenoid meningioma, low-grade carcinoma, or lymphoma. An endoscopic sphenoid sinus biopsy identified the lesion as a silent corticotroph pituitary neuroendocrine tumor/adenoma (SCA), confirmed by positive immunostaining for ACTH and T-PIT. The patient underwent a successful transsphenoidal resection, followed by a transient postoperative central adrenal insufficiency and diabetes insipidus, which resolved within eight months. Eighteen months postoperatively, the patient retains normal pituitary function with minimal residual tumor. This case illustrates the diagnostic challenge posed by SCAs when presenting as sphenoid sinus masses and highlights the importance of considering SCAs in similar cases.

## Introduction

Frontal headaches are a frequent clinical complaint, often attributed to common causes such as tension headaches, sinusitis, or migraines.^1^ In rare instances, however, these headaches can signal more complex underlying pathologies. Pituitary neuroendocrine tumors (PitNETs, formerly known as adenomas),^2^ benign tumors originating from epithelial cells in the adenohypophysis, are the most common tumors of the sellar region and typically present with either endocrine dysfunction or mass effect on nearby structures such as the optic chiasm and cranial nerves.^3–5^ Although larger pituitary tumors are less common, they may extend into adjacent areas such as the cavernous and sphenoid sinuses, leading to atypical symptoms that mimic sinus disease.^6^ This overlap can delay diagnosis, as nonspecific symptoms such as frontal headaches are more commonly linked to routine conditions.

Among PitNETs, a rare subset known as silent corticotroph adenomas (SCAs) produces adrenocorticotropic hormone (ACTH) without causing clinical signs of hypercortisolism.^7^ SCAs account for approximately 3%–6% of all PitNETs and about 5.5% of nonfunctioning PitNETs.^8,9^ Typically, SCAs are confirmed postoperatively through histopathological analysis, with immunohistochemical testing showing positive staining for ACTH or the pituitary-restricted T-box transcription factor (T-PIT), which helps distinguish SCAs from other nonfunctional PitNETs.^10^

In this report, we discuss a unique case of a patient with persistent frontal headaches who, after a thorough investigation, was diagnosed with an SCA manifesting as a mass in the right sphenoid sinus. Magnetic resonance imaging (MRI) played a critical role in this diagnosis, providing detailed visualization of the sellar and parasellar regions to assess the tumor’s origin and its impact on adjacent structures.

## Case report

A 43-year-old man of Turkish origin presented to his primary care physician with a 2-year history of frequent frontal headaches radiating to his occiput, occurring several times a week. His past medical history was notable for obstructive sleep apnea syndrome. His surgical history was unremarkable. His family history was negative for head and neck malignancy, PitNETs, Multiple Endocrine Neoplasia (MEN) syndrome, or any endocrinopathy. He is an active smoker, consuming one pack of cigarettes per day since age 19, reports social alcohol consumption, and denies recreational drug use. He does not take any regular medications. The patient denied symptoms suggestive of acromegaly, including acral enlargement, coarsening of facial features, and hyperhidrosis. He also denied symptoms indicative of hypogonadism, hypothyroidism, adrenal insufficiency, or hypercortisolism, including skin thinning, easy bruising, central weight gain, red or purple striae on his abdomen, plethoric facies, proximal muscle weakness. He denied galactorrhea, visual field defects, polyuria, polydipsia, or nocturia. On physical examination, the patient appeared well without acromegalic or Cushingoid features. His blood pressure was 139/101 mmHg, his heart rate was 70 bpm and his weight was 100 kg. Visual field testing by confrontation and extraocular movements were normal. Physical examination was unremarkable otherwise. No pertinent endocrine laboratory investigations had been performed prior to his referral.

**Figure 1.**
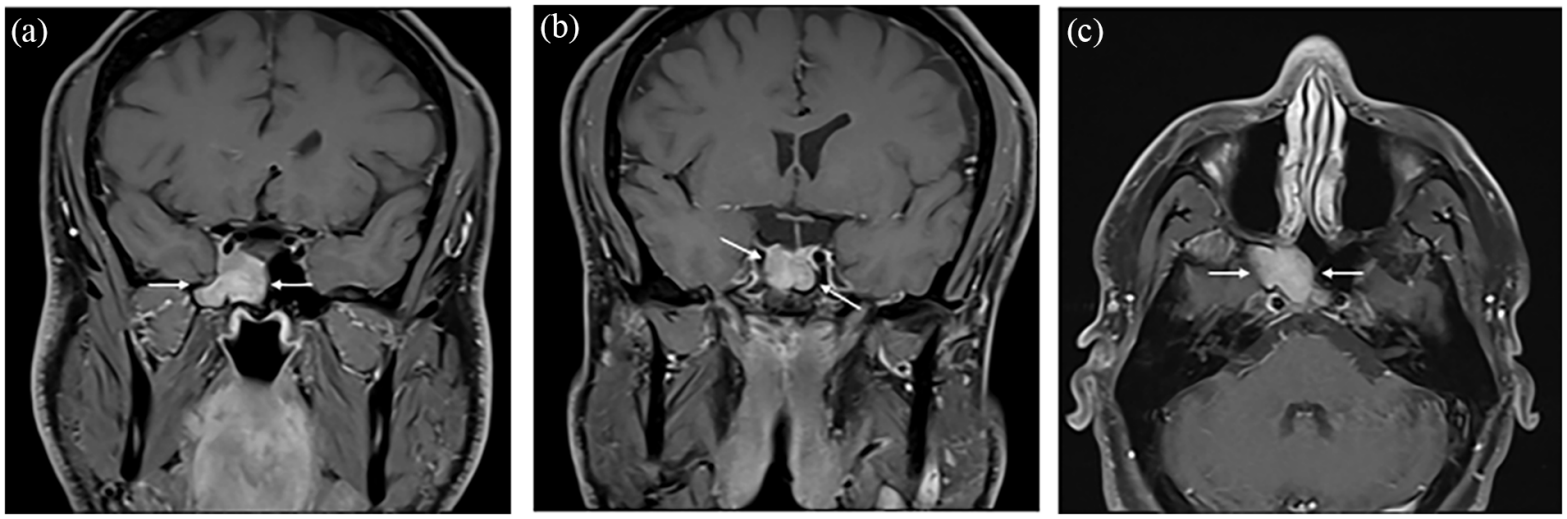
Preoperative MRI. Coronal (a, b) and axial (c) T1 post-gadolinium MRI showing enhancing mass involving the right inferolateral sella and extending into the right lateral recess of the sphenoid sinus as well as the cavernous sinus. MRI, magnetic resonance imaging.

In September 2022, the patient underwent an MRI of the paranasal sinuses, prompting referral to an otolaryngologist/head and neck surgeon. MRI was reported as: Right sphenoid sinus mass measuring 30 × 20 mm with right cavernous sinus extension, 180° encasement of the internal right carotid artery without a decrease in its caliber. The lesion posteriorly invades the sella turcica and cannot be distinguished from the pituitary gland. There is a mild deviation of the pituitary stalk toward the left. The lesion entirely occupies the sella turcica and it is iso- to hypointense in T2 (Figure 1). The lesion has nonspecific signal characteristics. Differential diagnosis includes an atypical sphenoid meningioma, low-grade carcinoma, or lymphoma; a hemangioma or cystic adenoid tumor is less likely.

Subsequently, the patient underwent an endoscopic biopsy of the sphenoid sinus mass under general anesthesia. Histopathological analysis was reported as: PitNET positive for ACTH/T-PIT / GATA-3, suggestive of a sparsely granulated corticotroph adenoma. Following these findings, the patient was referred to a multidisciplinary pituitary clinic at a tertiary university hospital, where he was evaluated by an endocrinologist and a neurosurgeon with expertise in transsphenoidal surgery. The patient completed a 24-h-urine collection for free cortisol to screen for hypercortisolism (Result: 284 nmol/d [ normal range 28–276 nmol/d ] but considered clinically significant when twice the upper normal) and a full pituitary panel revealed only a possible GH deficiency, based on the low IGF-1 levels. (Table 1). However, given that the patient was largely asymptomatic, no further endocrine assessment (including dynamic testing) was ordered for the possible somatotropic deficiency. Ideally, as per current Endocrine Society guidelines,^11^ two 24-hour urine-free cortisol collections, or alternative tests (one 1 mg dexamethasone suppression test or two late-night salivary cortisol tests) should have been completed to rule out biochemical hypercortisolism effectively. Unfortunately, in this patient’s case, given time and logistical constraints, it could not be done on time preoperatively.

**Table 1.** Pituitary hormone levels at presentation and follow-up.

Hormone	Level at presentation	12 months postoperatively	Normal range
IGF-1	13.8 nmol/L	12.5 nmol/L	14.8–42.7 nmol/L
LH	1 U/L	3.4 U/L	2–9 U/L
FSH	3 U/L	3.4 U/L	1–12 U/L
Total testosterone AM	9.32 nmol/L	15.9 nmol/L	8.30–33.00 nmol/L
TSH	2.090 mU/L	3.07 mU/L	0.270–4.200 mU/L
Free T4	—	17.1 pmol/L	9.0–26.0 pmol/L
ACTH	14.7 pmol/L	11.0 pmol/L	1.6–13.9 pmol/L
AM cortisol	373 nmol/L	309 nmol/L	119–618 nmol/L
Prolactin	8 mcg/L	6.9 mcg/L	4–15 mcg/L

**Figure 2.**
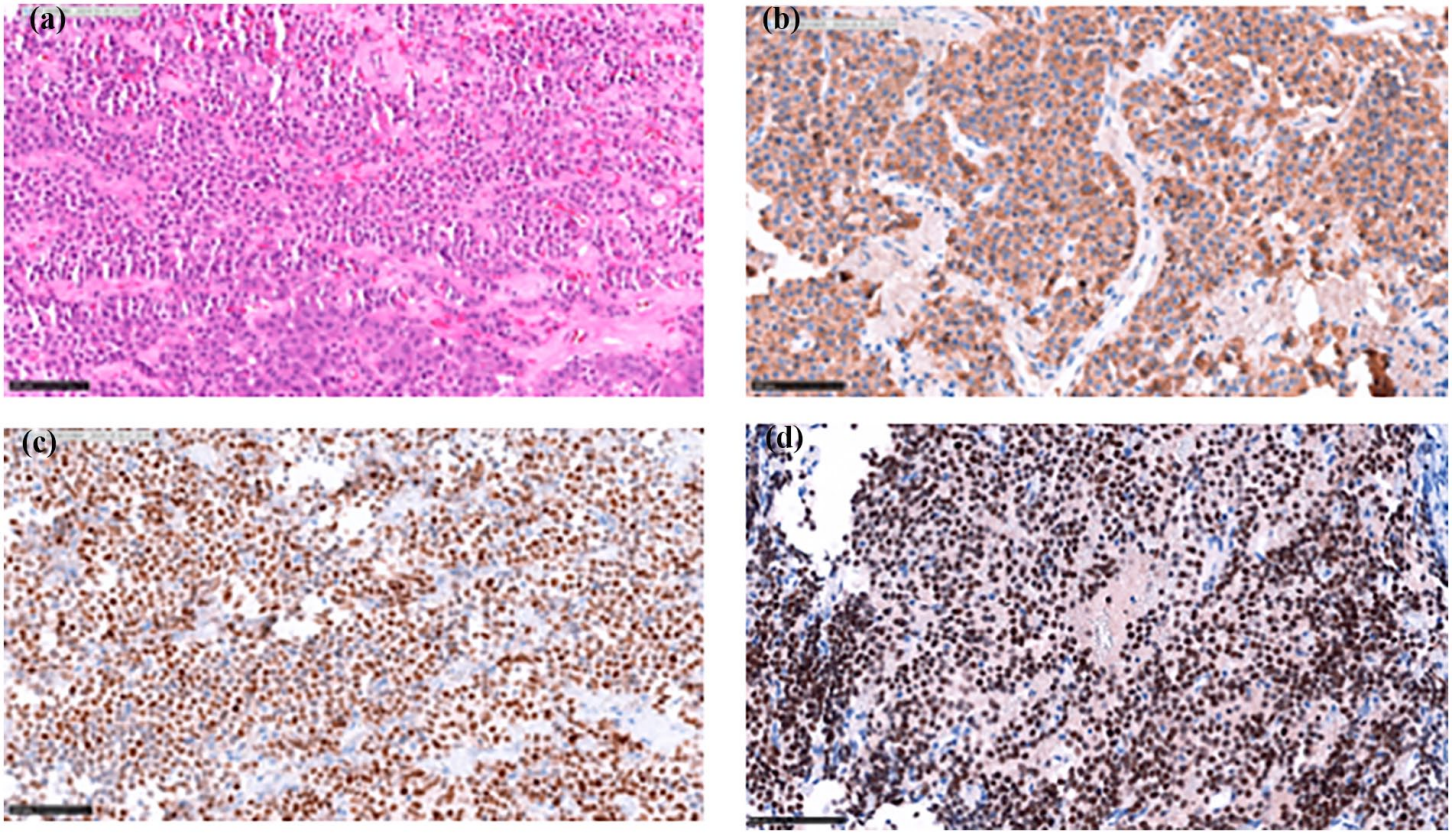
Histopathology of the corticotroph pituitary neuroendocrine tumor (adenoma). Sections from formalin-fixed paraffin-embedded (FFPE) tissue show hematoxylin and eosin staining (a), highlighting the epithelial-like tumoral proliferation composed of a homogeneous cell population without acinar architecture, with areas of collagenization and adjacent normal pituitary gland. Immunohistochemical staining for ACTH (b) demonstrates diffuse cytoplasmic positivity, confirming corticotroph lineage. Nuclear staining for T-PIT (c) shows strong nuclear positivity, supporting the diagnosis of a corticotroph adenoma. Staining for GATA-3 (d) demonstrates positive immunoreactivity, reflecting a subset of SCAs that co-express both GATA-3 and T-PIT, indicative of their mixed corticotroph and gonadotroph transcriptomic signature. Magnification 200×.

The preoperative MRI of the Sella Turcica demonstrated a 23 × 21 × 24 mm sellar lesion invading the right cavernous sinus and the right sphenoid sinus without suprasellar extension. Mild leftward deviation of the pituitary stalk was observed with no involvement of the left cavernous sinus. The patient underwent transsphenoidal surgery in April 2023. Final surgical pathology was reported as: epithelial-like tumoral proliferation composed of a homogeneous cell population without acinar architecture. Areas of collagenization are seen and some areas of the normal pituitary gland are also present. The tumor is seen just below the respiratory-type epithelium lining the mucosa. The tumoral cells are positive for T-PIT, ACTH, and GATA-3 (Figure 2). They are negative for PIT-1, SF-1, prolactin, GH, FSH, and LH. CK8/18 is positive with a strong cytoplasmic expression. The proliferation index (Ki-67 [measured with QuPath Instanseg embedding-based instance segmentation algorithm]) is 0.9%. The overall postoperative diagnosis confirmed the initial biopsy: corticotroph PitNET.

**Figure 3.**
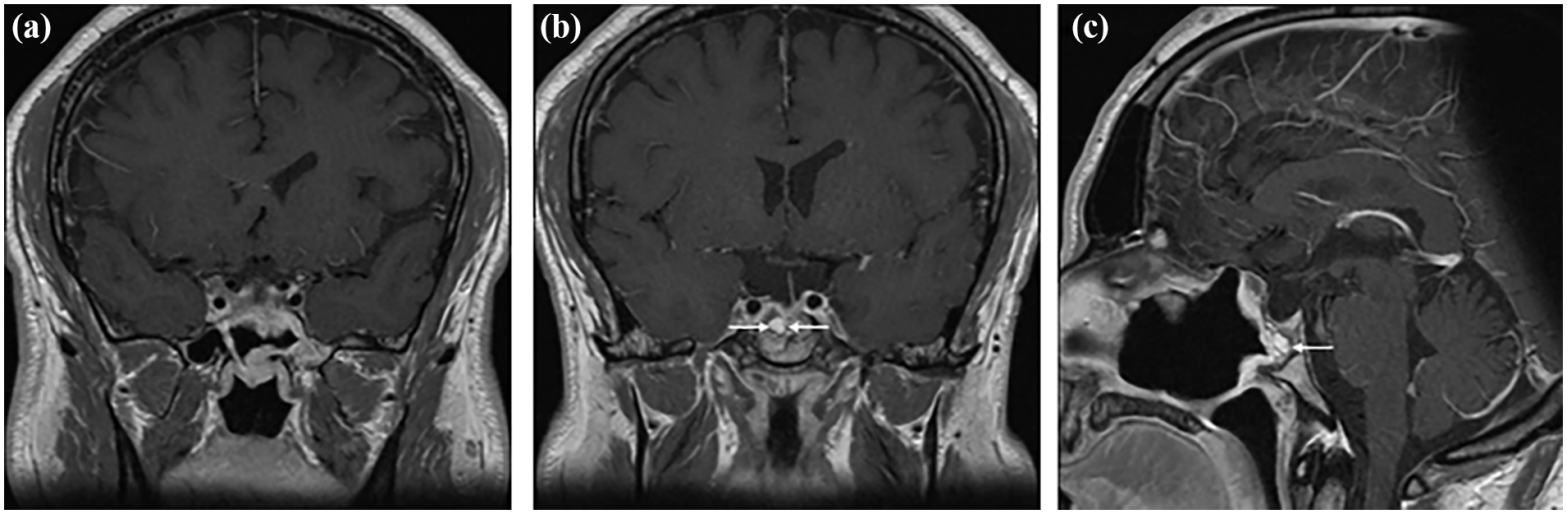
Postoperative MRI. Coronal (a, b) T1 post-gadolinium MRI showing resection of the tumor including the extension into the cavernous sinus. Sagittal reconstruction (c) demonstrating nasoseptal flap reconstruction of the sella.

The postoperative course was uneventful, however the patient developed transient central adrenal insufficiency and central diabetes insipidus requiring temporary treatment with hydrocortisone and desmopressin, which were successfully tapered over the following eight months. An ACTH Stimulation test three months postoperatively showed a peak cortisol of 701 nmol/L at 60 min, therefore ruling out residual central adrenal insufficiency and supporting hydrocortisone tapering. An overnight water deprivation test 7 months postoperatively showed a serum sodium of 141 mmol/L, a serum osmolality of 289 mOsm/kg, and a urine osmolality of 665 mOsm/kg, therefore ruling out central diabetes insipidus and allowing discontinuation of desmopressin. At eighteen months postoperatively, the patient maintains a normal anterior and posterior pituitary function, with a normal pituitary hormonal panel (Table 1). Follow-up MRI shows only a small residual tumor signal in the right cavernous sinus around the internal carotid artery, measuring 4 × 15 mm (Figure 3). He reported partial improvement of his headaches postoperatively.

## Discussion

SCAs are well documented for their aggressive growth patterns compared with other nonfunctional PitNETs. Recent studies demonstrated that SCAs have a higher predilection for invading bony and adjacent structures, such as the cavernous sinus and sphenoid sinus when compared to other PitNETs.^12,13^ These findings align with our case, where the tumor extended from the sella turcica into the sphenoid sinus and the right cavernous sinus encasing the internal carotid artery. This invasive growth pattern made surgical resection particularly challenging and emphasized the necessity of careful, multidisciplinary surgical management.

Of note, GATA-3 has emerged in recent years as a supplementary immunohistochemical marker for PitNET classification. A subset of SCAs express both GATA-3 and T-PIT, underlying their mixed corticotroph and gonadotroph transcriptomic signature.^14^ This highlights the importance of advanced immunohistochemical techniques in accurately classifying these tumors and guiding treatment strategies.

Very few similar cases have been reported to date. In a case series of 16 SCA patients by Zheng et al.^15^ who eventually developed overt Cushing syndrome, two cases presented with sphenoid sinus invasion and two other cases with simultaneous cavernous and sphenoid sinus invasion. In the same stream of thought, this case by Bashir et al.^16^ described a patient with a larger SCA invading the cavernous sinus and nasal cavity. Although the patient had no clinical features of Cushing syndrome, his biochemical workup showed more significant hypercortisolism and early recurrence postoperatively. The demographic characteristics were similar to our patient’s.

Headache is a common, nonspecific symptom in pituitary tumors, with younger age and a high Ki-67 index (>3%) correlating with more disabling preoperative headaches, likely reflecting tumor aggressiveness and invasiveness.^17^ Our patient’s young age aligns with this finding; however, the tumor Ki-67 index was lower than 1%. Transsphenoidal surgery often improves headache symptoms, although some patients may experience no improvement or develop new headaches, potentially affecting their quality of life.

Our case uniquely highlights the rare presentation of an SCA invading the sphenoid sinus and associated with persistent frontal headaches. While the headaches revealed the sinus mass, their exact relationship to the tumor remains uncertain, though some improvement in symptoms post-surgery suggests a possible contributing role. Such atypical presentations may delay diagnosis, especially if initial imaging studies are misinterpreted, or if primary headache disorders or sinusitis, are considered more likely. Several aspects in our case are rather unique and contrast with other reported cases, namely the lack of classic pituitary-related symptoms (e.g., visual disturbances or signs of hypercortisolism) and particularly the unusual sequence of events where the endoscopic sinus mass biopsy eventually led to the diagnosis of SCAs.

This case underscores the critical need to consider SCAs in the differential diagnosis of atypical sphenoid sinus masses, particularly in patients presenting with nonspecific symptoms such as chronic headaches. Due to their silent hormonal profile, SCAs frequently evade early detection, and diagnosis relies on the combination of advanced imaging with detailed histopathological analysis. Early recognition is essential as SCAs exhibit more aggressive behavior and higher recurrence rates compared to other pituitary adenomas, necessitating a proactive management approach. Collaboration among endocrinologists, otolaryngologists, neurosurgeons, radiologists, and pathologists was critical in achieving a successful outcome for this patient.

## Conclusion

PitNETs account for rare cases of sphenoid sinus masses and can present with nonspecific symptoms, such as headache. SCAs are a rare, but aggressive subtype of PitNETs, which are more likely to invade bony and adjacent structures. Ultimately, this report highlights the value of a multidisciplinary approach in the management of complex PitNETs invading the sphenoid sinus.

## Statements and Declarations

**Ethical considerations:** Our institution does not require ethical approval for reporting individual cases or case series.

**Data availability:** In this document, all image data presented have undergone rigorous privacy protection measures and have been obtained with the informed consent of the relevant patients. We solemnly pledge to adhere to ethical and privacy principles at all times, ensuring the lawful and compliant use of image data.

**Informed consent:** Written informed consent was obtained from the patient(s) for their anonymized information to be published in this article.
